# For Better Municipal Meat-inspection

**Published:** 1898-01

**Authors:** 


### MUNICIPAL MEAT-INSPECTION.

### FOR BETTER MUNICIPAL MEAT-INSPECTION.

AN important meeting was held in Philadelphia on December 6, 1897, in the interests of an improvement on the prevailing system existing in this city. The meeting was held under the auspices of the Woman's Health Protective Association, and presided over by Mrs. John H. Scribner, president of the organization. In opening the meeting, the president expressed very earnestly the interests of their organization in this movement. She referred to the work of their sister organization in New York, that had accomplished many reforms there, of the visit made by a committee of the Philadelphia association to a number of the small private slaughter-houses, and the deplorable conditions found there, and pledged the aid of their organization in every way possible to remedy the existing evils.

Dr. D. E. Salmon, Chief of the Bureau of Animal Industry, was then introduced. He spoke of the great work being done by this Bureau in meat-inspection for export and inter-state trade; of how exacting our foreign customers had become in this direction, and the good results that followed at home, and touched upon some of the important diseases for which protection was needed, and urged its need and importance all over the land.

Prof. R. S. Huidekoper, of New York, was then introduced, and, as one of those who for years had fully appreciated the inefficiency of the service in Philadelphia, urged its improvement. He spoke with much feeling as to the dangers, and referring to the system existing in Continental cities, he contrasted it with that prevalent in American cities, and forcibly pointed out the importance of this movement, and urged all present to interest themselves in improving the service.

Dr. H. D. Gill, Dean of the New York College of Veterinary Surgeons, was the next speaker, and the many dangers incidental to the preparation and care of meat for food were lucidly explained, and the many avenues of danger for its injury and of becoming an unwholesome food-product prejudicial to public health were very forcibly portrayed by the speaker. The duties of those charged with municipal affairs were pointedly referred to, and he hoped the movement inaugurated in Philadelphia would be successful and spread to many other cities.

Dr. A. W. Clement, of Baltimore, State Veterinarian of Maryland, in his remarks, spoke of the importance of this subject from a State point of view, and referred to the early attention given to attain these ends by the Hebrews and other sects. He looked for good results from the general discussion and agitation of the subject, and felt that the attention of the National Government to this subject was of much importance in educating our people to its need. The question of compensation by the Government was one that made difficult the solution of the question, but he thought this should be no barrier to its adoption in every large city.

Dr. Benjamin Lee, Secretary of the State Board of Health of Pennsylvania, gave an interesting account of the continued interest of the State Board in wholesome and pure food-products. He said that they had long appreciated the need of reform in the direction of greater safeguards around our meat-supply, and that the present movement would have the hearty co-operation of the State authorities, and hoped that it might spread to every city and town of this Commonwealth, that the good results flowing therefrom would better aid the great ends which such bodies as he was officially connected with, planned and worked to secure.

Dr. George Strawbridge, of the Philadelphia County Medical Society, spoke forcibly of the many reforms that every good citizen should be interested in, that call for solution in municipal government; of Philadelphia's need of a pure water-supply, a better-guarded food-supply, especially of meat and milk. He referred to

ARMY MEDICAL LIBRARY
WASHINGTON, D. C. U. S. A.

several of the important diseases that annually increase the death-rate beyond proper proportions, which are largely the outcome of impure milk and unwholesome meat. How essential these products were to our people at every stage of life. He referred to the attention given this subject in foreign countries, and how many municipalities had made central abattoirs a source of income to their cities, and lightened the burdens of everyone at the same time.

Miss Pope, of the Woman's Health Protective Association, gave a graphic account of what she had seen on a tour of some of our small slaughter-houses. She spoke of their unfavorable location, of the unsanitary surroundings, the general lack of cleanliness that prevailed about them; and of the many features that made it impossible in such places to prepare meat-products fit for food. Her remarks were fully coincided in by others of the visiting committee, and each of the speakers urged the members and everyone in attendance to array themselves in the battle for reform in our meat-inspection service.

Dr. Leonard Pearson, Secretary of the State Live-stock Sanitary Board of Pennsylvania, touched upon many features of this all-important subject; how great a flesh-consuming nation we are; how imperfect our city inspection-service is generally, save in New Orleans, La., and Montgomery, Ala.; how thoroughly this work is carried on abroad, and what centralized abattoirs have done for the producers and consumers in these cities, and, contrasting it with Philadelphia's imperfect system, made everyone feel how imperative was the need of prompt and thorough reform in our city.

Dr. A. C. Abbott, of the Health Department of the city, in speaking of the needed reform, said it was of the greatest importance and should receive the very earnest support of every good citizen.

Dr. W. Horace Hoskins closed the consideration of the subject by congratulating Philadelphia, in that the city possessed so aggressive a body of earnest, working women as the Woman's Health Protective Association was composed of; pointing with pride and due appreciation of the reforms they had already accomplished, and others they were leading to victory, all in the welfare and interest of every soul in this great community, and that he knew full well that their organization and its members, now pledged to further this movement, would be a tower of strength in adding this as an accomplished reform as another trophy upon their proud record of earnest, aggressive work in every good and beneficial movement for our people.

